# Vitamin D and Sex Differences in COVID-19

**DOI:** 10.3389/fendo.2020.567824

**Published:** 2020-09-30

**Authors:** Maria Teresa Pagano, Daniela Peruzzu, Anna Ruggieri, Elena Ortona, Maria Cristina Gagliardi

**Affiliations:** Center for Gender Specific Medicine, Istituto Superiore di Sanità (ISS), Rome, Italy

**Keywords:** coronavirus disease 2019, vitamin D, estrogen, inflammation, sex/gender differences

## Introduction

Hypovitaminosis D is implicated in various inflammatory, infectious and autoimmune diseases and recent lines of evidence suggest that it may represent a risk factor also for the ongoing epidemic of coronavirus disease 2019 (COVID-19) (1–3). In fact, the outcome of COVID-19 appears to be influenced by vitamin D status of populations (4, 5).

Several studies have clearly shown that 1,25(OH)2 vitamin D(3) (Vitamin D3, the active metabolite of vitamin D), besides its classical function in calcium dependent bone homeostasis, is actively involved in the regulation of innate and adaptive immune responses (6). In particular, it plays a key role in the control of the cytokine storm, i.e., the sudden acute increase in circulating levels of different pro-inflammatory cytokines, induced in several inflammatory conditions and also in COVID-19 (7). This activity of Vitamin D3 is carried out by inhibiting the production of the pro-inflammatory cytokines such as tumor necrosis factor- α (TNF-α) and interferon-γ (IFN-γ) but, also, by increasing the expression of the anti-inflammatory cytokine interleukin-10 (IL-10). Moreover, Vitamin D3 enhances the production of antimicrobial peptides such as human cathelicidin, LL-37 and defensins in several infections (8). A further important feature of Vitamin D3 is its capacity to reduce the risk of viral infections maintaining the integrity of the epithelium by the upregulation of genes which encode proteins required for tight, gap and adherens junctions (6). Further studies will be necessary to clarify whether all these anti-microbial effects could also occur against SARS-CoV-2, assigning to Vitamin D3 a protective role against COVID-19. Notably, Vitamin D3 enhances the expression of human angiotensin-converting enzyme 2 (ACE2), the functional receptor for SARS-CoV-2 (9, 10). ACE2 plays a protective role in acute respiratory distress syndrome and higher levels of ACE2 seem to be associated with better outcomes for lung diseases and, in particular, for COVID-19 (11–13).

Based on these considerations, in COVID-19 the inter-individual variability in circulating levels of 25-hydroxyvitamin D (25(OH)D), the biomarker of vitamin D status could be involved in the different severity of pulmonary inflammation and viral pathogenicity among individuals (14). Supporting the important protective role of Vitamin D3 in COVID-19 outbreak, negative correlations between mean levels of Vitamin D3 of European countries and the number of COVID-19 cases were observed (1, 13). Moreover, the lethality rate increased with age and with chronic disease comorbidity, both of which are associated with decreased vitamin D3 levels (15).

## COVID-19 and Sex Differences

To note, epidemiological data indicate that COVID-19 has a significantly higher lethality in men than in women (ratios up to 3:1), suggesting the presence of sex-dependent biological factors underlying these differences in disease outcome (16, 17). It is known that, in general, innate and acquired immune responses are more intense in females than in males (18). This can provide women with a more effective defense to fight new and infective pathogens, favoring viral clearance. Another significant explanation for sex differences in COVID‐19 lethality is the sex–dependent modulation of cellular receptors and co‐receptors used by SARS‐CoV‐2 to enter human host cells. In particular, ACE2 is encoded on X-chromosome, in sites commonly escaping the inactivation of one X-chromosome in mammalian XX cells (XCI), and could therefore be overexpressed in women (19). Moreover, estrogen induces an increase of ACE2 expression that, as reported above, could play a protective role in acute respiratory distress (11, 20), whereas androgen can increase the expression and activation of transmembrane serine-protease 2, (TMPRSS2), that facilitates virus-cell membrane fusion, thus favoring the infection (21).

Moreover, a large number of COVID-19 patients exhibit severe cardiovascular damage and those with pre-existing cardiovascular diseases appear to have an increased risk of death (22). To note, estrogen has known protective effects on the cardiovascular system mediated by estrogen receptors, resulting in the activation of endothelial nitric oxide synthase. Moreover, estrogen modulates serum lipoprotein and triglyceride levels and influences the expression of coagulant and fibrinolytic proteins. These estrogen-mediated actions could represent a further reason for the sex-specific differences in the outcome of COVID-19 (23, 24).

## COVID-19 and Sex Differences: A Role for Vitamin D3

A further interesting point is represented by the potential differences in serum level of 25(OH)D among men and women. Sanghera and co-workers (15) observed a significantly reduced level of 25(OH)D in both men and women with obesity that represents a further important risk factor for COVID-19. In this study, 25(OH)D level remains consistently lower in obese men than in obese women (15). On the contrary, in another study, Mucogiuri and co-workers (25) stratifying the sample population according to sex and body mass index (BMI), found that 25(OH)D concentrations were significantly higher in males compared to females in all BMI classes and decreased along with the increase of BMI values. Although these contrasting data seem to not assign to 25(OH)D a clear role in determining sex differences in obese COVID-19 patients, we think that attention could be paid to 25(OH)D levels in the context of this comorbidity.

Interestingly, sex differences have been observed in the immunomodulatory and anti-inflammatory effects of Vitamin D3 in some autoimmune diseases. In particular, a study of Correale and co-workers (26) showed that Vitamin D3 induces a stronger inhibition of the production of pro-inflammatory cytokines and a higher increase of anti-inflammatory cytokines in lymphocytes from multiple sclerosis female patients in comparison to those from male patients. Interestingly, Spanier and co-workers (27) suggested that Vitamin D3 acts in an estrogen-dependent manner in controlling T regulatory cell differentiation. Moreover, estrogen seems to increase the expression of the nuclear vitamin D receptor (VDR) gene in CD4+ T cells (28) and to decrease the expression of CYP24A1, the cytochrome P450 component of the 25-hydroxyvitamin D(3)-24-hydroxylase enzyme which inactivates Vitamin D3. In turn, Vitamin D3 exerts tissue-specific effects on peripheral estrogen metabolism (29). Hence, the sex-related immunomodulatory effects of Vitamin D3 suggest that it is possible to speculate that also in COVID-19, Vitamin D3 could play a role in the outcome and lethality.

## Conclusions

In conclusion, the outcome of COVID-19 appears to be influenced by the interaction among genetic, hormonal and environmental factors. The low levels of 25(OH)D could represent a risk factor for development of disease. In particular, it is tempting to hypothesize that the synergy between Vitamin D3 and estrogen could affect the sex differences in the outcome of patients with COVID-19. However, further studies will be mandatory in order to investigate the efficacy of Vitamin D3 supplements, in combination or not with estrogen agonists, as a valid adjuvant for prevention and/or treatment of this severe infectious disease.

## Author Contributions

MP and DP: study conception and design, and manuscript drafting. AR: critical revision. EO and MG: study conception and design, and critical revision. All authors contributed to the article and approved the submitted version.

## Conflict of Interest

The authors declare that the research was conducted in the absence of any commercial or financial relationships that could be construed as a potential conflict of interest.
